# Transversal Double‐Level Mandibular Distraction by Basal Bone Hinge Anchorage and Dentoalveolar Acrylic Plate for a Curved Distraction: Evolution of the Technique in a Double‐Jaw Distraction Case Report

**DOI:** 10.1155/crid/3447784

**Published:** 2026-07-05

**Authors:** Antonio Cortese, Francesca Spirito, Pier Paolo Claudio, Lorenzo Lo Muzio, Osvaldo Basilone, Franco Carlino

**Affiliations:** ^1^ Department of Clinical and Experimental Medicine, University of Foggia, Foggia, Italy, unifg.it; ^2^ Department of Pharmacology and Toxicology, University of Mississippi Medical Center, Jackson, Mississippi, USA, umc.edu; ^3^ Department of Surgery, Section of Maxillofacial Surgery, “Villa dei Pini” Hospital, Civitanova Marche, Macerata, Italy

## Abstract

Transverse mandibular deficit is characterized by dental crowding, retroalveolism, and vertical dystopia of the anterior alveolar process. Traditional treatments can cause periodontal, occlusion, and esthetic complications. Distraction osteogenesis represents a technique for enlarging bone segments and promoting the formation of regenerated bone that, after maturation, may show characteristics comparable to native bone, as reported in the literature. The study presents an evolution of a previously described technique, in which dentoalveolar distraction is achieved in a curvilinear shape that matches the natural morphology of the alveolar bone. The patient presented with severe dental arches crowding and a nasal breathing impairment. The surgical approach involved an incomplete Le Fort I osteotomy and the insertion of a hybrid distractor with four orthodontic bands for dental anchorage and four single‐ring plates for bone screw anchorage. On the anterior mandible, a double‐level distraction by vertical osteotomy at the symphysis basal bone level for immediate widening, connected with a stepwise osteotomy up to the alveolar bone at the osteotomy site, between the lateral incisor and canine, was performed. An acrylic device with a curved jackscrew, designed to ensure precise curved alveolar bone distraction while preserving intercondylar distance, was secured with dental wire ligatures. Postoperative evaluation revealed no complications. The present case suggests that the technique may be applied to address transverse mandibular deficiency. Within the limitations of a single case report, this approach appears to be technically feasible and may represent a promising modification of previously described techniques. Further studies are required to assess reproducibility and clinical outcomes.

## 1. Introduction

Dental crowding, retroalveolism, and vertical dystopia of the anterior alveolar process resulting in incongruent curves of Spee are the major signs of a transverse mandibular deficit [1–3]. Dental compensation, dental extraction, teeth slicing, and orthodontic arch expansion are the traditional and more conservative treatment options for transverse mandibular width deficit [4, 5]. However, these therapies are known to cause periodontal complications as well as occlusal or esthetic impairment [3, 6, 7].

Current orthodontic treatments often include dental extractions to balance dentoalveolar discrepancies and to reduce the risk of periodontal disease associated with nonextraction alignments treated with frontal teeth proclination in cases showing alveolar bone deficiency. This is particularly relevant in the anterior region, where the buccal wall is very thin with high esthetic relevance. In this esthetic area, a uniform level of the periodontal parabolas is paramount, as it conveys youthfulness, also reflecting a favorable genetic asset [8].

On the other hand, extractive treatments can lead to alveolar and dental arches collapse, negatively affecting the face and smile esthetics. For this reason, orthodontic and surgical therapy should aim to correct teeth crowding without extraction, solving dentoalveolar imbalance by increasing alveolar bone dimensions without reducing dental arches by extractions, also resulting in easier and safer orthodontic treatments [9, 10].

Modern therapy planning often involves the use of bone distraction techniques to enlarge the basal and alveolar bones and resolve dental crowding. Distraction osteogenesis is widely used to increase bone volume and correct skeletal deficiencies, allowing progressive expansion of bone structures with simultaneous adaptation of the surrounding soft tissues. Experimental and clinical evidence suggests that, following consolidation and remodeling, the newly formed bone may achieve structural and functional characteristics comparable to native bone [11–15].

Mandibular symphyseal distraction osteogenesis (MSDO) is a surgical technique aimed at increasing mandibular width through controlled bone expansion, providing a potential alternative to extractive orthodontic approaches in selected adult patients [16]. This technique, which involves the rapid expansion of the mandible, was first described in 1990 and can provide a surgical alternative to extractive orthodontic treatments for adult patients [17]. Although the effectiveness of MSDO is still being improved, various devices are available for the distraction process [18].

Mandibular dysmorphisms may involve discrepancies at different anatomical levels, including basal bone, alveolar bone, and dental structures. Effective correction often requires a combined approach addressing each component [19]. To achieve this type of correction, a technique has been developed that consists of an immediate transverse expansion of the mandibular basal bone and concurrent progressive distraction at the dentoalveolar level with final orthodontic refinement.

Mandibular bone distraction techniques have been limited in their spread due to encumbrance in the frontal esthetic area and the frequent contamination and infection of the devices due to the transmucosal anchorage components with contamination from oral cavity bacteria [20]. In order to avoid these transmucosal components, a technique was developed in which the transverse expansion of the mandible was divided into a double‐level technique: immediate expansion after mandibular osteotomy of the basal bone through fixation with plates and screws acting like hinges and progressive distraction of the dentoalveolar component using jackscrews on dental anchorage devices [19].

This technique, already published in previous articles [19], has been progressively modified to achieve not only a transverse increase but also a sagittal increase.

In this work, we aim to describe a further evolution of the technique, in which the dentoalveolar distraction is obtained in a curvilinear shape conforming to the natural morphology of the dentoalveolar arch in the frontal sector.

Unlike maxillary transversal distraction, where a bodily movement is the ideal goal, in mandibular transversal distraction, a curved activation device is necessary to preserve the intercondylar distance during distraction via a fan movement with the two condyles acting as hinges.

## 2. Materials and Methods

This case report represents a technique evolution after the already published case series in previous articles [19].

As a single case report, this study does not aim to provide statistical validation or comparative evaluation of outcomes, but rather to illustrate a technical modification and its clinical application. Indication for this surgical technique was as follows: teeth crowding for a transverse and sagittal dentoalveolar mandibular discrepancy in adult patients over 18; patients who also had transverse maxillary hypoplasia were also considered, as well as an association with another skeletal malocclusion (i.e., skeletal Class II, skeletal Class III, anterior open bite, and skeletal mandibular asymmetry). Exclusion criteria were systemic diseases including mental illnesses and neoplastic pathologies beyond an ongoing or completed treatment with bisphosphonates or other antiresorptive drugs, previous surgical treatment except for surgically assisted rapid palatal expansion (SARPE), bilateral sagittal split osteotomy, genioplasty, and Le Fort I osteotomy.

**Figure 1:**
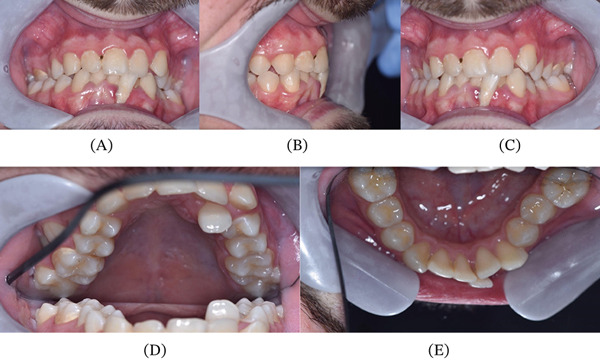
Preoperative view showing heavy crowding on both dental arches. (A) Frontal view, right side. (B) Lateral view. (C) Frontal view, left side. (D) Occlusal view of the upper jaw. (E) Occlusal view of the lower jaw.

The patient was referred to our service for severe dental arches crowding with transverse maxillary and mandibular constriction at the basal bone level, nasal breathing impairment, Class I occlusion, and asymptomatic TMJ function (Figure 1). The patient also refused teeth extractions for orthodontic treatment.

The study was conducted in accordance with the ethical principles of the Declaration of Helsinki and the principles of good clinical practice. The reported case was treated in 2021 within an institutional ethical framework (IRB Protocol No. 38/06). The patient provided written informed consent for both the surgical procedure and the use of clinical data for scientific purposes. Clinical data and measurements were collected from the patient the first time preoperatively (T0) and 1 year postoperatively (T1). On these occasions, an x‐ray orthopantomography, lateral frontal x‐ray cephalograms, and dental model measurements were carried out.

**Figure 2:**
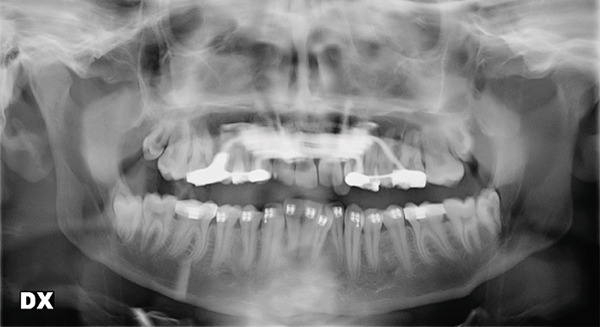
X‐rays showing a Hyrax distractor with four bands and four single‐ring plates.

Dental measurements were collected using an electronic caliper to evaluate transversal and sagittal expansion. For transverse expansion, the mandibular intercanine distance, the interpremolar distance of the mandibular first and second premolars (buccal and lingual cusps), and the intermolar distance of the mandibular first molars (mesiolingual and mesiobuccal cusps) were measured. For sagittal expansion, the line connecting the mesiobuccal cusps of the first molars to the interdental papilla between the central mandibular incisors was measured. The preservation of alveolar bone peaks was investigated using x‐ray panoramic images at T0 and T1. TMJ function was also analyzed in terms of TMJ pain, clicking on motion, and mouth opening limitation. An x‐ray image investigated possible condylar resorption (Figure 2).

**Figure 3:**
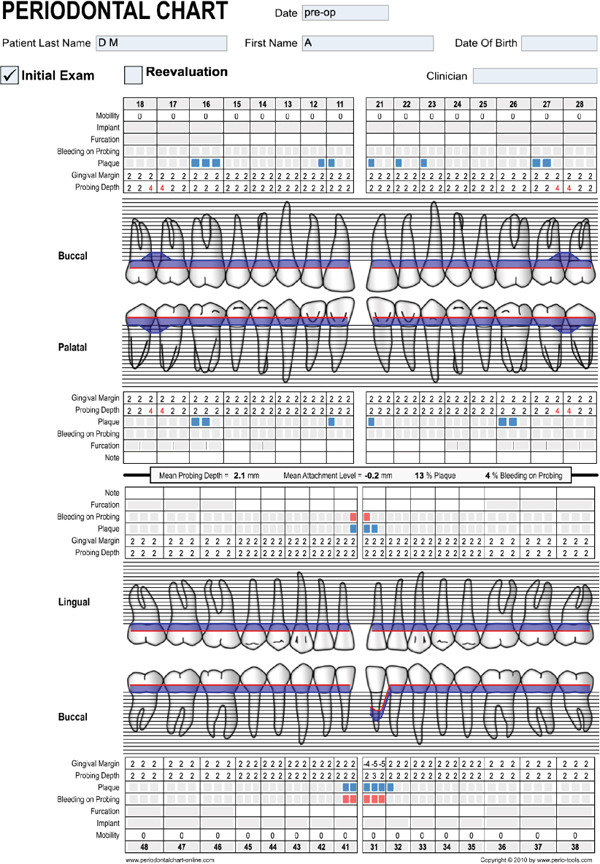
Preoperative periodontal chart.

A comprehensive periodontal examination was carried out, including the compilation of a periodontal chart with standard periodontal indices (plaque index, gingival index, probing pocket depth [PPD], and clinical attachment level [CAL]). These parameters were recorded at baseline and subsequently used to monitor periodontal health, with particular focus on Tooth 3.1, where improvement was expected following the procedure (Figure 3).

Prior to the surgery, orthodontic appliances were utilized to prepare for the procedure without the need for orthodontic alignment at this stage by a passive orthodontic arch wire.

### 2.1. Surgical Procedures

The surgical procedure was conducted while the patient was under general anesthesia and through rhinotracheal intubation.

The surgical procedure started with an incomplete Le Fort I osteotomy and the insertion of a bone‐ and tooth‐borne distractor equipped with a titanium jackscrew, as described in our previous work [19]. After ordinary Le Fort I osteotomies without down fracture at the anterolateral maxillae walls, pterygomaxillary, and septum base disjunction, a Hyrax distractor with four bands and four single‐ring plates was applied (Figure 2).

The titanium jackscrew arms were cast to four orthodontic bands on the upper first molars and first bicuspids for dental anchorage; bone anchorage was obtained using four self‐locking screws inserted into four single‐ring plates cast to the titanium jackscrew at a paramedian palatal site.

The screw insertion was directed backward and medially through the oral cavity in the basal bone septum crest site, where a sufficient amount of bone for screw insertion and sagittal osteotomy was available.

The mandibular surgery started with an incision in the buccal mucosa between Teeth 33 and 43 below the muscle insertion level for easier wound suture and healing. Once the periosteum was reached, it was detached from the incision site up to the lower alveolar bone level and downward up to the lower border of the mandible using a periosteal elevator. Osteotomy was then carried out on the anterior mandible, with a vertical osteotomy at the symphysis basal bone level connected with a stepwise osteotomy up to the lower level of the alveolar bone at the osteotomy site, between the lateral incisor and canine (42 and 43).

Osteotomies at the alveolar bone level were conducted by a tunneling technique up to the interdental septum, thus avoiding periosteum and fibromucosa elevation at this site.

**Figure 4:**
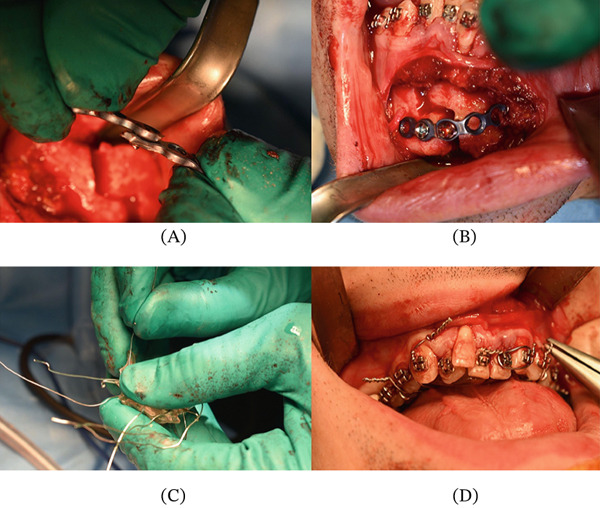
Intraoperative view. (A) Two plates connected by a single screw at the two overlapped medial holes, acting as a hinge. The screw was fixed at the posterior plate hole and free to rotate into the anterior plate hole. (B) The two plates are fixed at the symphysis basal bone for immediate widening. (C) Acrylic plate wire fixation at the first premolar and molar lower teeth. (D) Wire fixation of the lower teeth at the osteotomy site before distraction.

The osteotomies were carried out using thin rotating burs, as well as a thin chisel for final mobilization, particularly at the interdental alveolar septum. In this way, bone peak preservation and bone fragment nourishment were achieved, minimizing the risk of gingival clefts on adjacent teeth. Once the osteotomies were completed and the fragments mobilized, the two mandibular segments were immediately widened at the basal bone level using the symphysiotomy cut, resulting in an instant enlargement of the bone up to the planned amount. As a result, the two hemimandibular fragments were inclined lingually, keeping them in touch at the alveolar bone level by wire ligature on the brackets of the two adjacent teeth at the osteotomy site (Figure 4). Two osteosynthesis plates connected at the median site by a single screw acting like a hinge were fixed onto the two mandibular halves by four screws (two for each side).

The two plates were bent in an S‐shape on the sagittal plane at the last median holes to maintain a symmetrical position of the two mandibular halves after the last holes overlap at the median site. The last holes were connected by a single screw inserted in both plate holes; the posterior one was then fixed to the screw by hole compression with pliers. The screw head inserted into the last hole of the anterior plate was maintained free to rotate, acting like a hinge.

In this way, the two mandibular halves were able to rotate on the frontal plane during distraction by teeth‐borne plate activation without any vertical movement.

**Figure 5:**
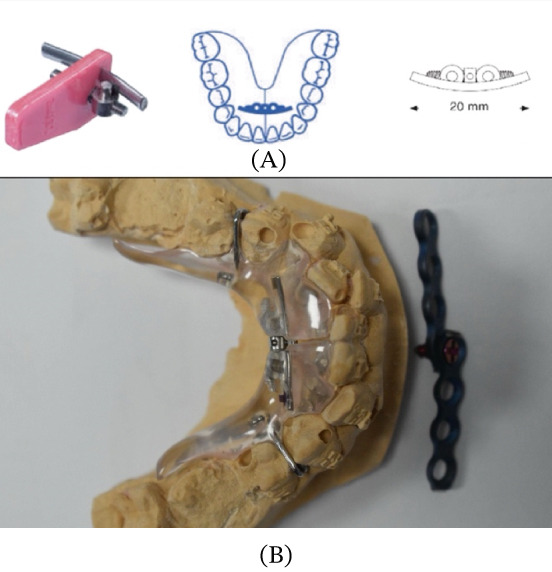
(A) Acrylic device with curved jackscrew for precise curved alveolar bone distraction preserving intercondylar distance, combined with (B) double plates with sagittal hinge for vertical stability at the basal bone level.

For this purpose, an acrylic device with a curved jackscrew for precise curved alveolar bone distraction preserving intercondylar distance was fixed with dental wire ligatures to the mandibular first molars and premolars (Figure 5).

To prevent the intercondylar distance from increasing during activation, trimming of the acrylic plate distal portion at the first molar and the bicuspid level was performed. This represented the active distraction device.

One week after the surgery, active transverse distraction was executed, and the screw was daily activated for a one‐quarter turn, resulting in a 0.20 mm distraction four times each day for the number of days needed to achieve sufficient transversal enlargement. In the reported case, activation was carried out for 8 days. The patient then underwent a 4‐month period of contention. After that, an orthodontic treatment was carried out for progressive alignment and resolution of the crowding, also with repositioning of the 31.

## 3. Results

**Figure 6:**
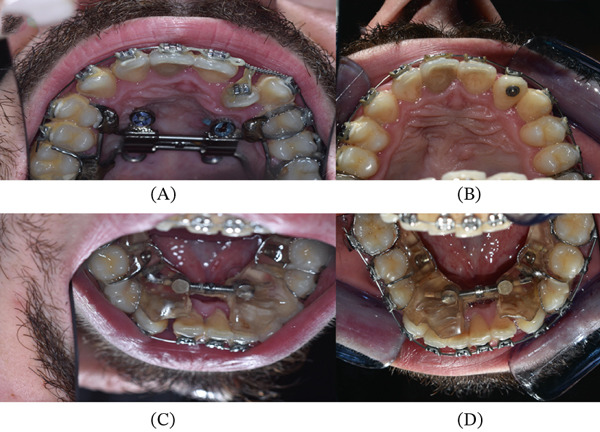
Postoperative view. (A, C) Occlusal view of the upper and lower jaw at T0 after surgery. (B, D) Occlusal view of the upper and lower jaw at T1, 1 year after surgery.

**Figure 7:**
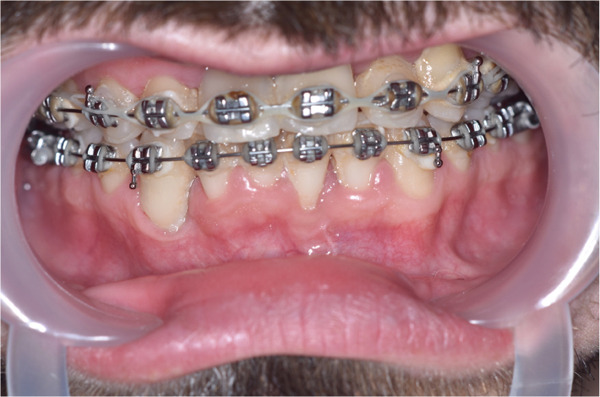
Postoperative views showing peak preservation and periodontal improvement in Class I and crossbite resolution.

Postoperative views are shown in Figures 6 and 7. No complications due to infection, decubitus, and preternatural mobility of the teeth were found in the postoperative period. The results of the expansion are shown in Table 1.

**Table 1:** Dental measurements of the patient at T0 (preoperatively, before orthodontic appliance positioning) and T1 (follow‐up, 1 year after surgery).

	T0	T1
Intercanine (mm)	23	30
Interpremolar buccal/first premolars (mm)	33	39
Interpremolar buccal/second premolars (mm)	38	47
Intermolar mesiobuccal/first molars (mm)	47	51
First molar mesiobuccal (papilla 1–1)	35	38

**Figure 8:**
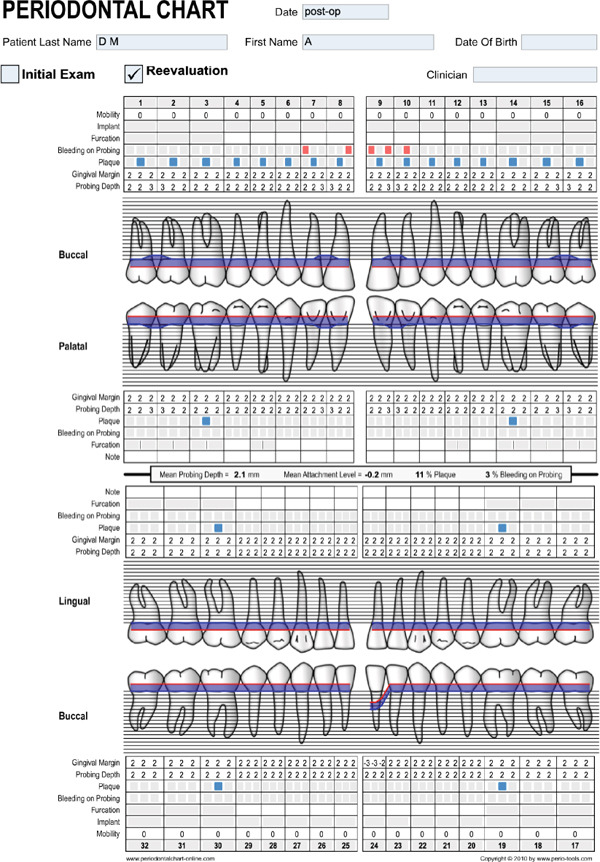
Postoperative periodontal chart.

Despite the presence of risk factors such as smoking and suboptimal oral hygiene, no major postoperative complications were observed. Periodontal parameters at Tooth 3.1 showed an improvement at follow‐up. From a periodontal point of view, specifically for Tooth 3.1, baseline measurements showed gingival margins of −4/−5 mm and probing depths of 2–3 mm, resulting in a mean CAL of ~7 mm. At the postoperative re‐evaluation, gingival margins improved to −2/−3 mm, and probing depths were uniformly 2 mm, corresponding to a mean CAL of ~4.7 mm. Overall, a clinical attachment gain of about 2–3 mm was observed, with normalization of probing depths and periodontal stabilization (Figure 8). However, it is not possible to determine whether this improvement is directly related to the surgical procedure or to other local or systemic factors.

## 4. Discussion

In this study, we presented a further evolution of the dentoalveolar distraction technique, aiming to achieve a curvilinear shape of the expansion that conforms to the natural morphology of the dentoalveolar arch in the frontal sector. This advancement not only simplifies the procedure but also may contribute to improved periodontal conditions in selected cases. By utilizing both transverse and sagittal components during the bone distraction process, new bone formation occurs, resulting in an alveolar bone morphology that is radiographically consistent with newly formed bone. This morphology is suitable for accommodating the roots of crowded teeth through subsequent orthodontic treatment with torque coil springs on a rectangular arch wire.

Unlike maxillary distraction, where a bodily movement is the ideal goal, mandibular distraction requires the use of a curved activation device to preserve the intercondylar distance during distraction. The device activation facilitates a fan movement with the two condyles acting as hinges, allowing for controlled distraction in a curvilinear manner.

The mandibular distraction technique differs from maxillary distraction in terms of the ideal goals and anchorage systems. While a bodily movement is sought after in maxillary distraction, it is contraindicated in the mandible due to the insertion of the condyles into the glenoid cavities, which restricts expansion. Therefore, we proposed a curvilinear distraction approach in the mandible.

In previous studies, mandibular transversal and sagittal distraction was performed in two stages, whereas in this case, we directly achieved curvilinear dentoalveolar expansion.

Additionally, we performed the alveolar osteotomy between 42 and 43 because of a wider interdental bone septum availability in this case, also achieving a stepwise osteotomy from basal bone up to alveolar bone levels for larger skeletal coping and better stability of the two stumps. Additionally, the intermolar expansion observed in the posterior sector should not be interpreted as a purely skeletal effect of distraction. In this case, orthodontic treatment contributed to molar torque correction and dental repositioning, resulting in an apparent increase in intermolar width. Therefore, the overall expansion reflects a combination of skeletal and dentoalveolar changes.

By implementing a double‐level technique for mandibular expansion, we could achieve immediate bone expansion at the basal bone level, which underwent robust healing without the risk of bone and soft tissue atrophy. Simultaneously, dentoalveolar distraction with the precise and conservative cutting of the interalveolar septum allowed for papilla preservation. This osteodistraction procedure preserved the periosteum and gum attachment at the alveolar bone level, as demonstrated in our previous work.

A comparable approach to mandibular distraction has been described by Bianchi et al. [21], who proposed a device‐based system for transverse mandibular expansion. While both techniques aim to address transverse mandibular deficiency, they differ in biomechanical design and activation pattern.

In the technique described by Bianchi et al. [21], the distraction is primarily guided by a rigid system, resulting in a predominantly linear expansion pattern. In contrast, the present approach introduces a curvilinear dentoalveolar distraction trajectory, intended to better reproduce the natural morphology of the dental arch and to maintain intercondylar distance through a fan‐like movement.

These biomechanical differences may influence the distribution of forces on periodontal structures and the overall pattern of expansion. However, given the limited evidence available and the absence of comparative studies, no definitive conclusions can be drawn regarding the relative advantages of one approach over the other.

Long‐term studies on mandibular distraction osteogenesis have reported heterogeneous stability outcomes, with relapse rates influenced by surgical protocol, biomechanical control, and patient‐related variables. Previous studies on mandibular distraction osteogenesis have reported variable long‐term stability, with follow‐up periods extending beyond 2–5 years. These findings suggest that treatment outcomes may depend on surgical technique, biomechanical control, fixation method, and patient‐related factors [22–24].

In the present case, the follow‐up period is limited to 1 year, which allows evaluation of short‐term outcomes but does not provide information on long‐term stability or relapse. By our hybrid maxillary distraction technique, a bodily movement of the two maxillary halves can be achieved, resulting in more effective and stable resolution of the posterior crossbite and maxillary constriction [19]. Breathing improvement was reported both in the Bologna case series and in our case, probably related to the basal bone widening of the jaws with more space for nasal airflow at the maxillary level and more space for the tongue at the mandibular level.

Another advantage in our technique was the absence of transmucosal anchoring devices, avoiding infection risks at the symphysis distraction site and second‐stage surgery for device removal.

Distraction osteogenesis is a biologically driven process of bone regeneration induced by controlled mechanical separation of bone segments. This process involves coordinated cellular and vascular responses that lead to progressive tissue formation and remodeling. Experimental and clinical evidence suggests that, after consolidation, the regenerated bone may achieve structural characteristics comparable to native bone [25, 26].

In our case, the distraction process was performed with minimal step enlargement, using four activations of 0.2 mm per day, resulting in a daily distraction rate of 0.8 mm, followed by strict stability gaps with rigid distraction device contention. Strict stability gaps were incorporated using a rigid distraction device system to ensure optimal bone healing. In this way, bone bridges within the initial bone–fibrous callus are broken during activation, followed by immediate new bone bridge healing via stabilization during activation intervals. The quality of bone resulting from this process is comparable to the native bone. This has significant implications for the periodontal health of teeth undergoing orthodontic movements in the regenerated bone area.

The biological process involved in the distraction procedure includes the recruitment of chemotactic and stem cells, which contribute to bone and soft tissue regeneration in the distracted segment. This distinguishes the distraction technique from other one‐step expansion techniques (segmented Le Fort I osteotomy with immediate widening) that involve bone and soft tissue segments, where expansion is achieved in a single step with large tissue gaps. Moreover, the soft tissue′s ability to regenerate and achieve segments identical to native ones can be attributed to the recruitment of chemotactic and stem cells from the surrounding tissues efficiently after the distraction procedure.

The results of this process can be observed in the excellent regeneration of bone, dental arches, gums, muscles, nerves, and skin, all of which closely resemble their native counterparts. This is particularly important for gum and skin areas, where traditional orthognathic treatments may result in insufficient tissue quality and thickness at the osteotomy sites.

Because maxillomandibular discrepancies may show different degrees of expression inside each jaw, to achieve ideal functional and esthetic results, three‐level planning is necessary for each jaw: (1) dental arches, (2) alveolar bone, and (3) basal bone.

Following new concepts for cleft lip/palate etiology showing neural crest migration impairment by a combination of genetic (CDH1 loss of function) and environmental (proinflammatory activation) factors [27], a lack of mesodermal migration after genetic asset might be advocated for maxillomandibular discrepancies and insufficiencies.

Specific surgical techniques are necessary to selectively correct each of the six possible discrepancy levels (three for each jaw) to achieve ideal results. This study has some limitations that should be considered when interpreting the findings. First, the report is based on a single clinical case and does not allow conclusions regarding generalizability or reproducibility of the proposed technique. Second, no histological or three‐dimensional radiological evaluation (e.g., CBCT‐based analysis) was performed, limiting the assessment of the biological characteristics of the regenerated bone. Third, measurements were obtained from plaster models using an electronic caliper, which does not provide a full three‐dimensional evaluation and may be subject to operator‐dependent variability.

Despite these limitations, the present case provides a detailed description of a technical modification and its clinical application, offering preliminary insights that may support further investigation in larger and controlled studies.

## 5. Conclusion

Within the limitations of a single case report, the proposed technique appears to be technically feasible and may represent a promising evolution of previously described mandibular distraction approaches.

**Figure 9:**
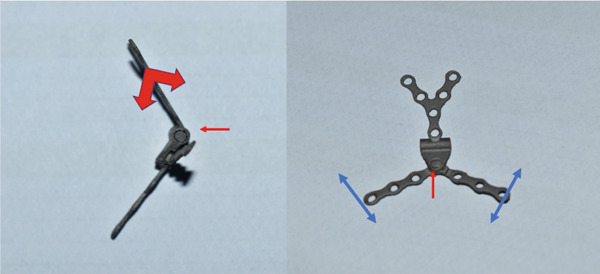
Triple‐plate combination with a transverse hinge for sagittal distraction and a sagittal hinge for transverse distraction. In the left panel, the red curved arrows indicate the sagittal distraction movement allowed by the transverse hinge, while the small red arrow marks the hinge. In the right panel, the blue arrows indicate the transverse distraction movement of the lateral plates, while the red arrow marks the sagittal hinge.

The proposed biomechanical configuration, including the triple‐plate system illustrated in Figure 9, represents a conceptual extension of the technique that requires further validation in future studies.

Further investigations are necessary to assess reproducibility, safety, and long‐term stability.

## Author Contributions

Conceptualization: A.C. and F.C.; methodology: A.C.; investigation: A.C.; data curation: A.C. and O.B.; formal analysis: F.S.; writing—original draft: A.C. and F.S.; writing—review and editing: A.C., F.S., and P.P.C.; validation: A.C., F.C., and P.P.C.; supervision: F.C. and L.L.M.

## Funding

Open access publishing facilitated by Universita degli Studi di Foggia, as part of the Wiley–CRUI‐CARE agreement.

## Disclosure

All authors have read and approved the final version of the manuscript. Prof. A.C. had full access to all of the data in this study and takes complete responsibility for the integrity of the data and the accuracy of the data analysis. The content is solely the responsibility of the authors and does not necessarily represent the official views of the National Institutes of Health.

## Conflicts of Interest

The authors declare no conflicts of interest.

## Data Availability

The authors confirm that the data supporting the findings of this study are available within the article.
